# Supplementary material for: Efficient Bayesian functional principal component analysis of irregularly-observed multivariate curves
Source: Comput Stat Data Anal. Author manuscript; Available in PMC 2026 Sep 5. (PMC7619451; doi:10.1016/j.csda.2024.108094)
Supplement: Supplementary file 1 [file EMS217722-supplement-Supplementary_material.pdf]

Supplementary material for: Efficient Bayesian functional principal component analysis of irregularly-observed multivariate curves

Tui H. Nolan[1,2], Sylvia Richardson[1], and Hélène Ruffieux*[1]

[1]MRC Biostatistics Unit, University of Cambridge
[2]University of Technology Sydney

Contents

A Proof of Proposition 4.1 **2**

B Exponential family form **2**

C Derivation of the multivariate functional principal component Gaussian likelihood fragment **3**

D Additional simulation results **6**
D.1 Comparison to a model specification with inferred score variances 6
D.2 Addendum on the accuracy of the variational algorithm 8
D.3 Addendum on the selection of K and L 9
D.4 Addendum on the comparison with frequentist multivariate FPCA 10
D.5 Addendum on the runtime profiling 11

Matrix algebraic background

We define the vec and vech operators, which are well-established (Gentle, 2007, e.g.). For a $d_1 \times d_2$ matrix, the vec operator concatenates the columns of the matrix from left to right. For a $d_1 \times d_1$ matrix, the vech operator concatenates the columns of the matrix after removing the above diagonal elements. For example, suppose that $\boldsymbol{A} = [\ (2,-3)^\intercal \quad (-1,1)^\intercal\ ]$. Then $\text{vec}(\boldsymbol{A}) = (2,-3,-1,1)^\intercal$ and $\text{vech}(\boldsymbol{A}) = (2,-3,1)^\intercal$. For a $d^2 \times 1$ vector $\boldsymbol{a}$, $\text{vec}^{-1}(\boldsymbol{a})$ is the $d \times d$ matrix such that $\text{vec}\{\text{vec}^{-1}(\boldsymbol{a})\} = \boldsymbol{a}$. Additionally, the matrix $\boldsymbol{D}_d$ is the duplication matrix of order d, and it is such that $\boldsymbol{D}_d \, \text{vech}(\boldsymbol{A}) = \text{vec}(\boldsymbol{A})$ for a $d \times d$ symmetric matrix $\boldsymbol{A}$. Furthermore, $\boldsymbol{D}_d^+ \equiv (\boldsymbol{D}_d^\intercal \boldsymbol{D}_d)^{-1} \boldsymbol{D}_d^\intercal$ is the Moore-Penrose inverse of $\boldsymbol{D}_d$, where $\boldsymbol{D}_d^+ \, \text{vec}(\boldsymbol{A}) = \text{vech}(\boldsymbol{A})$.

For a set of d matrices $\{\boldsymbol{M}_i\}_{i=1,\dots,d}$, we define:

$$\underset{i=1,\dots,d}{\text{stack}}(\boldsymbol{M}_i) \equiv \begin{bmatrix} \boldsymbol{M}_1 \\ \vdots \\ \boldsymbol{M}_d \end{bmatrix} \quad \text{and} \quad \underset{i=1,\dots,d}{\text{blockdiag}}(\boldsymbol{M}_i) \equiv \begin{bmatrix} \boldsymbol{M}_1 & \boldsymbol{O} & \cdots & \boldsymbol{O} \\ \boldsymbol{O} & \boldsymbol{M}_2 & \cdots & \boldsymbol{O} \\ \vdots & \vdots & \ddots & \vdots \\ \boldsymbol{O} & \boldsymbol{O} & \cdots & \boldsymbol{M}_d \end{bmatrix},$$

with the first of these definitions requiring that each $\boldsymbol{M}_i$ has the same number of columns.

*Corresponding author: helene.ruffieux@mrc-bsu.cam.ac.uk

A Proof of Proposition 4.1

First, set $\boldsymbol{\theta} \equiv \{\boldsymbol{\nu}, \boldsymbol{\zeta}_1, \ldots, \boldsymbol{\zeta}_n, \boldsymbol{\sigma}_\varepsilon^2, \boldsymbol{a}_\varepsilon, \boldsymbol{\sigma}_\mu^2, \boldsymbol{a}_\mu, \boldsymbol{\sigma}_{\psi_1}^2, \ldots, \boldsymbol{\sigma}_{\psi_L}^2, \boldsymbol{a}_{\psi_1}, \ldots, \boldsymbol{a}_{\psi_L}\}$, the entire parameter set. Then, the optimal posterior density function for $\boldsymbol{\nu}$ in (9) that minimises the Kullback–Leibler divergence of its left-hand side from its right-hand side is computed according to

$$q(\boldsymbol{\nu}) = C_1 \exp\left\{\mathbb{E}_{q(\boldsymbol{\theta}\setminus\boldsymbol{\nu})} \log p(\boldsymbol{x}, \boldsymbol{\theta})\right\}, \tag{A1}$$

where C_1 is a constant of proportionality. Next, note that

$$\log p(\boldsymbol{x}, \boldsymbol{\theta}) = \sum_{j=1}^{p} \left\{ \sum_{i=1}^{n} \log p(\boldsymbol{x}_i^{(j)} \mid \boldsymbol{\nu}^{(j)}, \boldsymbol{\zeta}_i, \sigma_\varepsilon^{(j)2}) + \log p(\boldsymbol{\nu}^{(j)} \mid \sigma_\varepsilon^{((j))2}) \right\} + f(\boldsymbol{\theta} \setminus \boldsymbol{\nu}), \tag{A2}$$

where $\boldsymbol{x}^{(j)} \equiv (\boldsymbol{x}_1^{(j)\top}, \ldots, \boldsymbol{x}_n^{(j)\top})^\top$, for $j = 1, \ldots, p$, and $f(\boldsymbol{\theta} \setminus \boldsymbol{\nu})$ represents an arbitrary function that does not depend on $\boldsymbol{\nu}$. Substituting (A2) into (A1), we have

$$q(\boldsymbol{\nu}) = C_2 \prod_{j=1}^{p} \exp\left[\mathbb{E}_{q(\boldsymbol{\theta}\setminus\boldsymbol{\nu}^{(j)})} \left\{ \sum_{i=1}^{n} \log p(\boldsymbol{x}_i^{(j)} \mid \boldsymbol{\nu}^{(j)}, \boldsymbol{\zeta}_i, \sigma_\varepsilon^{(j)2}) + \log p(\boldsymbol{\nu}^{(j)} \mid \sigma_\varepsilon^{((j))2}) \right\}\right] = \prod_{j=1}^{p} q(\boldsymbol{\nu}^{(j)}),$$

where C_2 is an arbitrary constant of proportionality.

B Exponential family form

We first describe the exponential family form for the normal distribution. For a $d \times 1$ multivariate normal random vector $\boldsymbol{x} \sim \mathrm{N}(\boldsymbol{\mu}, \boldsymbol{\Sigma})$, the probability density function of $\boldsymbol{x}$ can be shown to satisfy

$$p(\boldsymbol{x}) = \exp\left\{ \boldsymbol{T}_{\text{vec}}(\boldsymbol{x})^\top \boldsymbol{\eta}_{\text{vec}} - A_{\text{vec}}(\boldsymbol{\eta}_{\text{vec}}) - \frac{d}{2}\log(2\pi) \right\}, \tag{B3}$$

where $\boldsymbol{T}_{\text{vec}}(\boldsymbol{x}) \equiv \{\boldsymbol{x}^\top, \text{vec}(\boldsymbol{x}\boldsymbol{x}^\top)^\top\}^\top$ is the vector of sufficient statistics and $\boldsymbol{\eta}_{\text{vec}} \equiv (\boldsymbol{\eta}_{\text{vec},1}^\top, \boldsymbol{\eta}_{\text{vec},2}^\top)^\top \equiv [(\boldsymbol{\Sigma}^{-1}\boldsymbol{\mu})^\top, -\frac{1}{2}\{\text{vec}(\boldsymbol{\Sigma}^{-1})\}^\top]^\top$ is the natural parameter vector. The function $A_{\text{vec}}(\boldsymbol{\eta}_{\text{vec}}) = -\frac{1}{4}\boldsymbol{\eta}_{\text{vec},1}^\top\{\text{vec}^{-1}(\boldsymbol{\eta}_{\text{vec},2})\}^{-1}\boldsymbol{\eta}_{\text{vec},1} - \frac{1}{2}\log|-2\,\text{vec}^{-1}(\boldsymbol{\eta}_{\text{vec},2})|$ is the log-partition function. The inverse mapping of the natural parameter vector is (Wand, 2017, equation S.4)

$$\boldsymbol{\mu} = -\frac{1}{2}\left\{\text{vec}^{-1}(\boldsymbol{\eta}_{\text{vec},2})\right\}^{-1}\boldsymbol{\eta}_{\text{vec},1} \quad \text{and} \quad \boldsymbol{\Sigma} = -\frac{1}{2}\left\{\text{vec}^{-1}(\boldsymbol{\eta}_{\text{vec},2})\right\}^{-1}. \tag{B4}$$

We will refer to the representation of the multivariate normal probability density function in (B3) as the *vec-based representation*.

Alternatively, a more storage-economical representation of the multivariate normal probability density function is the *vech-based representation*:

$$p(\boldsymbol{x}) = \exp\left\{ \boldsymbol{T}_{\text{vech}}(\boldsymbol{x})^\top \boldsymbol{\eta}_{\text{vech}} - A_{\text{vech}}(\boldsymbol{\eta}_{\text{vech}}) - \frac{d}{2}\log(2\pi) \right\},$$

where the vector of sufficient statistics, the natural parameter vector and the log-partition function are, $\boldsymbol{T}_{\text{vech}}(\boldsymbol{x}) \equiv \{\boldsymbol{x}^\top, \text{vec}(\boldsymbol{x}\boldsymbol{x}^\top)^\top\}^\top$, $\boldsymbol{\eta}_{\text{vech}} \equiv (\boldsymbol{\eta}_{\text{vech},1}^\top, \boldsymbol{\eta}_{\text{vech},2}^\top)^\top \equiv [(\boldsymbol{\Sigma}^{-1}\boldsymbol{\mu})^\top, -\frac{1}{2}\boldsymbol{D}_d^\top\{\text{vec}(\boldsymbol{\Sigma}^{-1})\}^\top]^\top$ and $A_{\text{vech}}(\boldsymbol{\eta}_{\text{vech}}) = -\frac{1}{4}\boldsymbol{\eta}_{\text{vech},1}^\top\{\text{vec}^{-1}(\boldsymbol{D}_d^{+\top}\boldsymbol{\eta}_{\text{vech},2})\}^{-1}\boldsymbol{\eta}_{\text{vech},1} - \frac{1}{2}\log|-2\,\text{vec}^{-1}(\boldsymbol{D}_d^{+\top}\boldsymbol{\eta}_{\text{vech},2})|$, respectively. The inverse mapping of the natural parameter vector under the vech-based representation is

$$\boldsymbol{\mu} = -\frac{1}{2}\left\{\text{vec}^{-1}(\boldsymbol{D}_d^{+\top}\boldsymbol{\eta}_{\text{vech},2})\right\}^{-1}\boldsymbol{\eta}_{\text{vech},1} \quad \text{and} \quad \boldsymbol{\Sigma} = -\frac{1}{2}\left\{\text{vec}^{-1}(\boldsymbol{D}_d^{+\top}\boldsymbol{\eta}_{\text{vech},2})\right\}^{-1}. \tag{B5}$$

The other major distribution within the exponential family that is pivotal for this article is the inverse-χ^2 distribution. A random variable x has an inverse-χ^2 distribution with shape parameter $\xi > 0$ and scale parameter $\lambda > 0$ if the probability density function of x is

$$p(x) = \frac{(\lambda/2)^{\xi/2}}{\Gamma(\xi/2)} x^{-(\xi+2)/2} \exp\left(-\frac{\lambda}{2x}\right) \mathbb{I}(x > 0),$$

where the vector of sufficient statistics, the natural parameter vector and the log-partition function are $\boldsymbol{T}(x) \equiv (\log(x), 1/x)^{\mathsf{T}}$, $\boldsymbol{\eta} = (\eta_1, \eta_2)^{\mathsf{T}} = \{-\frac{1}{2}(\xi+2), -\frac{\lambda}{2}\}^{\mathsf{T}}$ and $A(\boldsymbol{\eta}) \equiv \log\{\Gamma(\xi/2)\} - \frac{\xi}{2}\log(\lambda/2)$, respectively. Note that $\Gamma(z) \equiv \int_0^\infty u^{z-1} e^u du$ is the gamma function, $\mathbb{I}(\cdot)$ is the indicator function, $\zeta > 0$ is the scale parameter and $\lambda > 0$ is the shape parameter. The inverse mapping of the natural parameter vector is $\xi = -2\eta_1 - 2$ and $\lambda = -2\eta_2$.

C Derivation of the multivariate functional principal component Gaussian likelihood fragment

From (8), we have, for $i = 1, \ldots, n$ and $j = 1, \ldots, p$,

$$\log p(\boldsymbol{x}_i^{(j)} | \boldsymbol{\nu}^{(j)}, \boldsymbol{\zeta}_i, \sigma_\varepsilon^{(j)2}) = -\frac{n_i^{(j)}}{2} \log(\sigma_\varepsilon^{(j)2}) - \frac{1}{2\sigma_\varepsilon^{(j)2}} \left|\left| \boldsymbol{x}_i^{(j)} - \boldsymbol{C}_i^{(j)} \left(\boldsymbol{\nu}_\mu^{(j)} + \sum_{l=1}^L \zeta_{il} \boldsymbol{\nu}_{\psi_l}^{(j)} \right) \right|\right|^2 + \text{const.} \qquad \text{(C6)}$$

First, we establish the natural parameter vector for each of the optimal posterior density functions. These natural parameter vectors are essential for determining expectations with respect to the optimal posterior distribution. From equation (10) of Wand (2017), we deduce that the natural parameter vector for $q(\boldsymbol{\nu}^{(j)})$, $j = 1, \ldots, p$ is

$$\boldsymbol{\eta}_{q(\boldsymbol{\nu}^{(j)})} = \boldsymbol{\eta}_{p(\boldsymbol{x}|\boldsymbol{\nu}, \boldsymbol{\zeta}_1, \ldots, \boldsymbol{\zeta}_n, \sigma_\varepsilon^{(1)2}, \ldots, \sigma_\varepsilon^{(p)2}) \to \boldsymbol{\nu}^{(j)}} + \boldsymbol{\eta}_{\boldsymbol{\nu}^{(j)} \to p(\boldsymbol{x}|\boldsymbol{\nu}, \boldsymbol{\zeta}_1, \ldots, \boldsymbol{\zeta}_n, \sigma_\varepsilon^{(1)2}, \ldots, \sigma_\varepsilon^{(p)2})},$$

the natural parameter vector for $q(\boldsymbol{\zeta}_i)$, $i = 1, \ldots, n$, is

$$\boldsymbol{\eta}_{q(\boldsymbol{\zeta}_i)} = \boldsymbol{\eta}_{p(\boldsymbol{x}|\boldsymbol{\nu}, \boldsymbol{\zeta}_1, \ldots, \boldsymbol{\zeta}_n, \sigma_\varepsilon^{(1)2}, \ldots, \sigma_\varepsilon^{(p)2}) \to \boldsymbol{\zeta}_i} + \boldsymbol{\eta}_{\boldsymbol{\zeta}_i \to p(\boldsymbol{x}|\boldsymbol{\nu}, \boldsymbol{\zeta}_1, \ldots, \boldsymbol{\zeta}_n, \sigma_\varepsilon^{(1)2}, \ldots, \sigma_\varepsilon^{(p)2})},$$

and the natural parameter vector for $q(\sigma_\varepsilon^{(j)2})$, $j = 1, \ldots, p$ is

$$\boldsymbol{\eta}_{q(\sigma_\varepsilon^{(j)2})} = \boldsymbol{\eta}_{p(\boldsymbol{x}|\boldsymbol{\nu}, \boldsymbol{\zeta}_1, \ldots, \boldsymbol{\zeta}_n, \sigma_\varepsilon^{(1)2}, \ldots, \sigma_\varepsilon^{(p)2}) \to \sigma_\varepsilon^{(j)2}} + \boldsymbol{\eta}_{\sigma_\varepsilon^{(j)2} \to p(\boldsymbol{x}|\boldsymbol{\nu}, \boldsymbol{\zeta}_1, \ldots, \boldsymbol{\zeta}_n, \sigma_\varepsilon^{(1)2}, \ldots, \sigma_\varepsilon^{(p)2})}.$$

Next, we consider the updates for standard expectations that occur for each of the random variables and random vectors in (C6). For each $\boldsymbol{\nu}^{(j)}$, we need to determine the mean vector $\mathbb{E}_q(\boldsymbol{\nu}^{(j)})$ and the covariance matrix $\mathbb{C}\text{ov}_q(\boldsymbol{\nu}^{(j)})$. The expectations are taken with respect to the normalisation of

$$m_{p(\boldsymbol{x}|\boldsymbol{\nu}, \boldsymbol{\zeta}_1, \ldots, \boldsymbol{\zeta}_n, \sigma_\varepsilon^{(1)2}, \ldots, \sigma_\varepsilon^{(p)2}) \to \boldsymbol{\nu}^{(j)}}(\boldsymbol{\nu}^{(j)}) \, m_{\boldsymbol{\nu}^{(j)} \to p(\boldsymbol{x}|\boldsymbol{\nu}, \boldsymbol{\zeta}_1, \ldots, \boldsymbol{\zeta}_n, \sigma_\varepsilon^{(1)2}, \ldots, \sigma_\varepsilon^{(p)2})}(\boldsymbol{\nu}^{(j)}),$$

which is a multivariate normal density function with natural parameter vector $\boldsymbol{\eta}_{q(\boldsymbol{\nu}^{(j)})}$. From (B4), we have

$$\begin{aligned} \mathbb{E}_q(\boldsymbol{\nu}^{(j)}) &\longleftarrow -\frac{1}{2} \left[\text{vec}^{-1} \left\{ \left(\boldsymbol{\eta}_{q(\boldsymbol{\nu}^{(j)})} \right)_2 \right\} \right]^{-1} \left(\boldsymbol{\eta}_{q(\boldsymbol{\nu}^{(j)})} \right)_1 \\ \text{and} \quad \mathbb{C}\text{ov}_q(\boldsymbol{\nu}^{(j)}) &\longleftarrow -\frac{1}{2} \left[\text{vec}^{-1} \left\{ \left(\boldsymbol{\eta}_{q(\boldsymbol{\nu}^{(j)})} \right)_2 \right\} \right]^{-1}. \end{aligned} \qquad \text{(C7)}$$

Furthermore, the mean vector has the form

$$\mathbb{E}_q(\boldsymbol{\nu}^{(j)}) \equiv \left\{ \mathbb{E}_q(\boldsymbol{\nu}_\mu^{(j)})^{\mathsf{T}}, \mathbb{E}_q(\boldsymbol{\nu}_{\psi_1}^{(j)})^{\mathsf{T}}, \ldots, \mathbb{E}_q(\boldsymbol{\nu}_{\psi_L}^{(j)})^{\mathsf{T}} \right\}^{\mathsf{T}}, \qquad \text{(C8)}$$

and the covariance matrix has the form

$$\mathbb{C}\text{ov}_q(\boldsymbol{\nu}) \equiv \begin{bmatrix} \mathbb{C}\text{ov}_q(\boldsymbol{\nu}_\mu^{(j)}) & \mathbb{C}\text{ov}_q(\boldsymbol{\nu}_\mu^{(j)}, \boldsymbol{\nu}_{\psi_1}^{(j)}) & \ldots & \mathbb{C}\text{ov}_q(\boldsymbol{\nu}_\mu^{(j)}, \boldsymbol{\nu}_{\psi_L}^{(j)}) \\ \mathbb{C}\text{ov}_q(\boldsymbol{\nu}_{\psi_1}^{(j)}, \boldsymbol{\nu}_\mu^{(j)}) & \mathbb{C}\text{ov}_q(\boldsymbol{\nu}_{\psi_1}^{(j)}) & \ldots & \mathbb{C}\text{ov}_q(\boldsymbol{\nu}_{\psi_1}^{(j)}, \boldsymbol{\nu}_{\psi_L}^{(j)}) \\ \vdots & \vdots & \ddots & \vdots \\ \mathbb{C}\text{ov}_q(\boldsymbol{\nu}_{\psi_L}^{(j)}, \boldsymbol{\nu}_\mu^{(j)}) & \mathbb{C}\text{ov}_q(\boldsymbol{\nu}_{\psi_L}^{(j)}, \boldsymbol{\nu}_{\psi_1}^{(j)}) & \ldots & \mathbb{C}\text{ov}_q(\boldsymbol{\nu}_{\psi_L}^{(j)}) \end{bmatrix}. \qquad \text{(C9)}$$

Similarly, for each $i = 1,\dots,n$, we need to determine the optimal mean vector and covariance matrix for $\boldsymbol{\zeta}_i$, which are $\mathbb{E}_q(\boldsymbol{\zeta}_i)$ and $\mathbb{C}\mathrm{ov}_q(\boldsymbol{\zeta}_i)$, respectively. The expectations are taken with respect to the normalisation of

$$m_{p(\boldsymbol{x}|\boldsymbol{\nu},\boldsymbol{\zeta}_1,\dots,\boldsymbol{\zeta}_n,\sigma_\varepsilon^{(1)2},\dots,\sigma_\varepsilon^{(p)2})\to\boldsymbol{\zeta}_i}(\boldsymbol{\zeta}_i)\, m_{\boldsymbol{\zeta}_i\to p(\boldsymbol{x}|\boldsymbol{\nu},\boldsymbol{\zeta}_1,\dots,\boldsymbol{\zeta}_n,\sigma_\varepsilon^{(1)2},\dots,\sigma_\varepsilon^{(p)2})}(\boldsymbol{\zeta}_i),$$

which is a multivariate normal density function with natural parameter vector $\boldsymbol{\eta}_{q(\boldsymbol{\zeta}_i)}$. According to (B5),

$$\begin{aligned}&\mathbb{E}_q(\boldsymbol{\zeta}_i) \longleftarrow -\frac{1}{2}\left[\mathrm{vec}^{-1}\left\{\boldsymbol{D}_L^{+\mathsf{T}}\left(\boldsymbol{\eta}_{q(\boldsymbol{\zeta}_i)}\right)_2\right\}\right]^{-1}\left(\boldsymbol{\eta}_{q(\boldsymbol{\zeta}_i)}\right)_1\\ \text{and}\quad &\mathbb{C}\mathrm{ov}_q(\boldsymbol{\zeta}_i) \longleftarrow -\frac{1}{2}\left[\mathrm{vec}^{-1}\left\{\boldsymbol{D}_L^{+\mathsf{T}}\left(\boldsymbol{\eta}_{q(\boldsymbol{\zeta}_i)}\right)_2\right\}\right]^{-1},\quad \text{for } i = 1,\dots,n.\end{aligned} \tag{C10}$$

Finally, for each $j = 1,\dots,p$, we need to determine $\mathbb{E}_q(1/\sigma_\varepsilon^{(j)2})$, with the expectation taken with respect to the normalisation of

$$m_{p(\boldsymbol{x}|\boldsymbol{\nu},\boldsymbol{\zeta}_1,\dots,\boldsymbol{\zeta}_n,\sigma_\varepsilon^{(1)2},\dots,\sigma_\varepsilon^{(p)2})\to\sigma_\varepsilon^{(j)2}}(\sigma_\varepsilon^{(j)2})\, m_{\sigma_\varepsilon^{(j)2}\to p(\boldsymbol{x}|\boldsymbol{\nu},\boldsymbol{\zeta}_1,\dots,\boldsymbol{\zeta}_n,\sigma_\varepsilon^{(1)2},\dots,\sigma_\varepsilon^{(p)2})}(\sigma_\varepsilon^{(j)2}).$$

This is an inverse-χ^2 density function, with natural parameter vector $\boldsymbol{\eta}_{q(\sigma_\varepsilon^{(j)2})}$. According to Result 6 of Maestrini and Wand (2021),

$$\mathbb{E}_q(1/\sigma_\varepsilon^{(j)2}) \longleftarrow \frac{\left(\boldsymbol{\eta}_{q(\sigma_\varepsilon^{(j)2})}\right)_1 + 1}{\left(\boldsymbol{\eta}_{q(\sigma_\varepsilon^{(j)2})}\right)_2}.$$

Now, we turn our attention to the derivation of the message passed from $p(\boldsymbol{x} \mid \boldsymbol{\nu},\boldsymbol{\zeta}_1,\dots,\boldsymbol{\zeta}_n,\sigma_\varepsilon^{(1)2},\dots,\sigma_\varepsilon^{(p)2})$ to $\boldsymbol{\nu}^{(j)}$, $j = 1,\dots,p$. Notice that

$$\boldsymbol{C}_i^{(j)}\left(\boldsymbol{\nu}_\mu^{(j)} - \sum_{l=1}^{L}\zeta_{il}\boldsymbol{\nu}_{\psi_l}^{(j)}\right) = (\widetilde{\boldsymbol{\zeta}}_i^{\mathsf{T}}\otimes\boldsymbol{C}_i^{(j)})\boldsymbol{\nu}^{(j)}. \tag{C11}$$

Therefore, as a function of $\boldsymbol{\nu}^{(j)}$, (C6) can be re-written as

$$\begin{aligned}\log p(\boldsymbol{x}_i^{(j)} \mid \boldsymbol{\nu}^{(j)},\boldsymbol{\zeta}_i,\sigma_\varepsilon^{(j)2}) &= -\frac{1}{2\sigma_\varepsilon^{(j)2}}\left|\left|\boldsymbol{x}_i^{(j)} - (\widetilde{\boldsymbol{\zeta}}_i^{\mathsf{T}}\otimes\boldsymbol{C}_i^{(j)})\boldsymbol{\nu}^{(j)}\right|\right|^2 + \text{terms not involving } \boldsymbol{\nu}^{(j)}\\ &= \begin{bmatrix}\boldsymbol{\nu}^{(j)}\\ \mathrm{vec}(\boldsymbol{\nu}^{(j)}\boldsymbol{\nu}^{(j)\mathsf{T}})\end{bmatrix}^{\mathsf{T}}\begin{bmatrix}\frac{1}{\sigma_\varepsilon^{(j)2}}(\widetilde{\boldsymbol{\zeta}}_i^{\mathsf{T}}\otimes\boldsymbol{C}_i^{(j)})^{\mathsf{T}}\boldsymbol{x}_i^{(j)}\\ -\frac{1}{2\sigma_\varepsilon^{(j)2}}\mathrm{vec}\left\{(\widetilde{\boldsymbol{\zeta}}_i\widetilde{\boldsymbol{\zeta}}_i^{\mathsf{T}})\otimes(\boldsymbol{C}_i^{(j)\mathsf{T}}\boldsymbol{C}_i^{(j)})\right\}\end{bmatrix}\\ &\quad + \text{terms not involving } \boldsymbol{\nu}^{(j)}.\end{aligned}$$

According to equation (8) of Wand (2017), the message from the factor $p(\boldsymbol{x} \mid \boldsymbol{\nu},\boldsymbol{\zeta}_1,\dots,\boldsymbol{\zeta}_n,\sigma_\varepsilon^{(1)2},\dots,\sigma_\varepsilon^{(p)2})$ to $\boldsymbol{\nu}^{(j)}$ is

$$m_{p(\boldsymbol{x}|\boldsymbol{\nu},\boldsymbol{\zeta}_1,\dots,\boldsymbol{\zeta}_n,\sigma_\varepsilon^{(1)2},\dots,\sigma_\varepsilon^{(p)2})\to\boldsymbol{\nu}^{(j)}}(\boldsymbol{\nu}^{(j)}) \propto \exp\left\{\begin{bmatrix}\boldsymbol{\nu}^{(j)}\\ \mathrm{vec}(\boldsymbol{\nu}^{(j)}\boldsymbol{\nu}^{(j)\mathsf{T}})\end{bmatrix}^{\mathsf{T}}\boldsymbol{\eta}_{p(\boldsymbol{x}|\boldsymbol{\nu},\boldsymbol{\zeta}_1,\dots,\boldsymbol{\zeta}_n,\sigma_\varepsilon^{(1)2},\dots,\sigma_\varepsilon^{(p)2})\to\boldsymbol{\nu}^{(j)}}\right\},$$

which is proportional to a multivariate normal density function. The update for the message's natural parameter vector, in (12), is dependent upon the mean vector and covariance matrix of $\widetilde{\boldsymbol{\zeta}}_i$, which are

$$\mathbb{E}_q(\widetilde{\zeta}_i) = \{1,\mathbb{E}_q(\boldsymbol{\zeta}_i)^{\mathsf{T}}\}^{\mathsf{T}} \quad \text{and} \quad \mathbb{C}\mathrm{ov}_q(\widetilde{\zeta}_i) = \mathrm{blockdiag}\left\{0,\mathbb{C}\mathrm{ov}_q(\boldsymbol{\zeta}_i)\right\},\quad \text{for } i = 1,\dots,n, \tag{C12}$$

where $\mathbb{E}_q(\boldsymbol{\zeta}_i)$ and $\mathbb{C}\mathrm{ov}_q(\boldsymbol{\zeta}_i)$ are defined in (C10). Note that a standard statistical result allows us to write

$$\mathbb{E}_q(\widetilde{\boldsymbol{\zeta}}_i\widetilde{\boldsymbol{\zeta}}_i^{\mathsf{T}}) = \mathbb{C}\mathrm{ov}_q(\widetilde{\boldsymbol{\zeta}}_i) + \mathbb{E}_q(\widetilde{\boldsymbol{\zeta}}_i)\,\mathbb{E}_q(\widetilde{\boldsymbol{\zeta}}_i)^{\mathsf{T}},\quad \text{for } i = 1,\dots,n. \tag{C13}$$

Next, notice that

$$\sum_{l=1}^{L} \zeta_{il} \boldsymbol{\nu}_{\psi_l}^{(j)} = \boldsymbol{V}_{\psi}^{(j)} \boldsymbol{\zeta}_i \tag{C14}$$

Then, for each $i = 1, \dots, n$, the log-density function in (C6) can be represented as a function of $\boldsymbol{\zeta}_i$ by

$$\begin{aligned}
\log p(\boldsymbol{x}_i^{(j)} \mid \boldsymbol{\nu}^{(j)}, \boldsymbol{\zeta}_i, \sigma_\varepsilon^{(j)2}) &= -\frac{1}{2\sigma_\varepsilon^{(j)2}} \left|\left| \boldsymbol{x}_i^{(j)} - \boldsymbol{C}_i^{(j)} \boldsymbol{\nu}_\mu^{(j)} - \boldsymbol{C}_i^{(j)} \boldsymbol{V}_\psi^{(j)} \boldsymbol{\zeta}_i \right|\right|^2 + \text{terms not involving } \boldsymbol{\zeta}_i \\
&= \begin{bmatrix} \boldsymbol{\zeta}_i \\ \text{vech}(\boldsymbol{\zeta}_i \boldsymbol{\zeta}_i^\top) \end{bmatrix}^\top \begin{bmatrix} \frac{1}{\sigma_\varepsilon^{(j)2}} (\boldsymbol{V}_\psi^{(j)\top} \boldsymbol{C}_i^{(j)\top} \boldsymbol{x}_i^{(j)} - \boldsymbol{h}_{\mu\psi,i}^{(j)}) \\ -\frac{1}{2\sigma_\varepsilon^{(j)2}} \boldsymbol{D}_L^\top \text{vec}(\boldsymbol{H}_{\psi,i}^{(j)}) \end{bmatrix} \\
&\quad + \text{terms not involving } \boldsymbol{\zeta}_i.
\end{aligned}$$

According to equation (8) of Wand (2017), the message from the factor $p(\boldsymbol{x} \mid \boldsymbol{\nu}, \boldsymbol{\zeta}_1, \dots, \boldsymbol{\zeta}_n, \sigma_\varepsilon^{(1)2}, \dots, \sigma_\varepsilon^{(p)2})$ to $\boldsymbol{\zeta}_i$ is

$$m_{p(\boldsymbol{x}|\boldsymbol{\nu},\boldsymbol{\zeta}_1,\dots,\boldsymbol{\zeta}_n,\sigma_\varepsilon^{(1)2},\dots,\sigma_\varepsilon^{(p)2}) \to \boldsymbol{\zeta}_i}(\boldsymbol{\zeta}_i) \propto \exp\left\{ \begin{bmatrix} \boldsymbol{\zeta}_i \\ \text{vech}(\boldsymbol{\zeta}_i \boldsymbol{\zeta}_i^\top) \end{bmatrix}^\top \boldsymbol{\eta}_{p(\boldsymbol{x}|\boldsymbol{\nu},\boldsymbol{\zeta}_1,\dots,\boldsymbol{\zeta}_n,\sigma_\varepsilon^{(1)2},\dots,\sigma_\varepsilon^{(p)2}) \to \boldsymbol{\zeta}_i} \right\},$$

which is proportional to a multivariate normal density function. The message's natural parameter vector update, in (13), is dependant on the following expectations that are yet to be determined:

$$\mathbb{E}_q(\boldsymbol{V}_\psi^{(j)}), \quad \mathbb{E}_q(\boldsymbol{H}_{\psi,i}^{(j)}), \quad \text{and} \quad \mathbb{E}_q(\boldsymbol{h}_{\mu\psi,i}^{(j)}), \quad i = 1, \dots, n \quad \text{and} \quad j = 1, \dots, p.$$

Now, we have,

$$\mathbb{E}_q(\boldsymbol{V}_\psi^{(j)}) = \left[\mathbb{E}_q(\boldsymbol{\nu}_{\psi_1}^{(j)}) \quad \dots \quad \mathbb{E}_q(\boldsymbol{\nu}_{\psi_L}^{(j)}) \right], \tag{C15}$$

where, for $l = 1, \dots, L$, $\mathbb{E}_q(\boldsymbol{\nu}_{\psi_l}^{(j)})$ is defined by (C7) and (C8). Next, $\mathbb{E}_q(\boldsymbol{h}_{\mu\psi,i}^{(j)})$ is an $L \times 1$ vector, with lth component being

$$\mathbb{E}_q(\boldsymbol{h}_{\mu\psi,i}^{(j)})_l = \text{tr}\{\mathbb{C}\text{ov}_q(\boldsymbol{\nu}_\mu^{(j)}, \boldsymbol{\nu}_{\psi_l}^{(j)}) \boldsymbol{C}_i^{(j)\top} \boldsymbol{C}_i^{(j)}\} + \mathbb{E}_q(\boldsymbol{\nu}_{\psi_l}^{(j)})^\top \boldsymbol{C}_i^{(j)\top} \boldsymbol{C}_i^{(j)} \mathbb{E}_q(\boldsymbol{\nu}_\mu^{(j)}), \quad l = 1, \dots, L, \tag{C16}$$

which depends on sub-vectors of $\mathbb{E}_q(\boldsymbol{\nu}^{(j)})$ and sub-blocks of $\mathbb{C}\text{ov}_q(\boldsymbol{\nu}^{(j)})$ that are defined in (C8) and (C9), respectively. Finally, $\mathbb{E}_q(\boldsymbol{H}_{\psi,i}^{(j)})$ is an $L \times L$ matrix, with (l, l') component being

$$\mathbb{E}_q(\boldsymbol{H}_{\psi,i}^{(j)})_{l,l'} = \text{tr}\{\mathbb{C}\text{ov}_q(\boldsymbol{\nu}_{\psi_{l'}}^{(j)}, \boldsymbol{\nu}_{\psi_l}^{(j)}) \boldsymbol{C}_i^{(j)\top} \boldsymbol{C}_i^{(j)}\} + \mathbb{E}_q(\boldsymbol{\nu}_{\psi_l}^{(j)})^\top \boldsymbol{C}_i^{(j)\top} \boldsymbol{C}_i^{(j)} \mathbb{E}_q(\boldsymbol{\nu}_{\psi_{l'}}^{(j)}), \quad l, l' = 1, \dots, L. \tag{C17}$$

The final message to consider is the message from $p(\boldsymbol{x} \mid \boldsymbol{\nu}, \boldsymbol{\zeta}_1, \dots, \boldsymbol{\zeta}_n, \sigma_\varepsilon^{(1)2}, \dots, \sigma_\varepsilon^{(p)2})$ to $\sigma_\varepsilon^{(j)2}$, for $j = 1, \dots, p$. As a function of $\sigma_\varepsilon^{(j)2}$, (C6) takes the form

$$\begin{aligned}
\log p(\boldsymbol{x}_i^{(j)} \mid \boldsymbol{\nu}^{(j)}, \boldsymbol{\zeta}_i, \sigma_\varepsilon^{(j)2}) &= -\frac{n_i^{(j)}}{2} \log(\sigma_\varepsilon^{(j)2}) - \frac{1}{2\sigma_\varepsilon^{(j)2}} \left|\left| \boldsymbol{x}_i^{(j)} - \boldsymbol{C}_i^{(j)} \boldsymbol{V}^{(j)} \widetilde{\boldsymbol{\zeta}}_i \right|\right|^2 \\
&\quad + \text{terms not involving } \sigma_\varepsilon^{(j)2} \\
&= \begin{bmatrix} \log(\sigma_\varepsilon^{(j)2}) \\ \frac{1}{\sigma_\varepsilon^{(j)2}} \end{bmatrix}^\top \begin{bmatrix} -\frac{n_i^{(j)}}{2} \\ -\frac{1}{2} \left|\left| \boldsymbol{x}_i^{(j)} - \boldsymbol{C}_i^{(j)} \boldsymbol{V}^{(j)} \widetilde{\boldsymbol{\zeta}}_i \right|\right|^2 \end{bmatrix} + \text{terms not involving } \sigma_\varepsilon^{(j)2}.
\end{aligned}$$

According to equation (8) of Wand (2017), the message from $p(\boldsymbol{x} \mid \boldsymbol{\nu}, \boldsymbol{\zeta}_1, \dots, \boldsymbol{\zeta}_n, \sigma_\varepsilon^{(1)2}, \dots, \sigma_\varepsilon^{(p)2})$ to $\sigma_\varepsilon^{(j)2}$ is

$$m_{p(\boldsymbol{x}|\boldsymbol{\nu},\boldsymbol{\zeta}_1,\dots,\boldsymbol{\zeta}_n,\sigma_\varepsilon^{(1)2},\dots,\sigma_\varepsilon^{(p)2}) \to \sigma_\varepsilon^{(j)2}}(\sigma_\varepsilon^{(j)2}) \propto \exp\left\{ \begin{bmatrix} \log(\sigma_\varepsilon^{(j)2}) \\ 1/\sigma_\varepsilon^{(j)2} \end{bmatrix}^\top \boldsymbol{\eta}_{p(\boldsymbol{x}|\boldsymbol{\nu},\boldsymbol{\zeta}_1,\dots,\boldsymbol{\zeta}_n,\sigma_\varepsilon^{(1)2},\dots,\sigma_\varepsilon^{(p)2}) \to \sigma_\varepsilon^{(j)2}} \right\},$$

which is proportional to an inverse-χ^2 density function. The message's natural parameter vector, in (14), depends on the mean of the square norm $\|\boldsymbol{x}_i^{()j} - \boldsymbol{C}_i^{(j)}\boldsymbol{V}^{(j)}\widetilde{\boldsymbol{\zeta}}_i\|^2$, for $i = 1,\ldots,n$. This expectation takes the form

$$\mathbb{E}_q\left(\left|\left|\boldsymbol{x}_i^j - \boldsymbol{C}_i^{(j)}\boldsymbol{V}^{(j)}\widetilde{\boldsymbol{\zeta}}_i\right|\right|^2\right) = \boldsymbol{x}_i^{(j)\intercal}\boldsymbol{x}_i^{(j)} - 2\,\mathbb{E}_q(\widetilde{\boldsymbol{\zeta}}_i)^\intercal\,\mathbb{E}_q(\boldsymbol{V}^{(j)})^\intercal\boldsymbol{C}_i^{(j)\intercal}\boldsymbol{x}_i^{(j)} + \mathrm{tr}\left[\left\{\mathbb{C}\mathrm{ov}_q(\widetilde{\boldsymbol{\zeta}}_i) + \mathbb{E}_q(\widetilde{\boldsymbol{\zeta}}_i)\,\mathbb{E}_q(\widetilde{\boldsymbol{\zeta}}_i)^\intercal\right\}\mathbb{E}_q(\boldsymbol{H}_i^{(j)})\right],$$

where we introduce the matrices

$$\boldsymbol{H}_i^{(j)} \equiv \begin{bmatrix} h_{\mu,i}^{(j)} & \boldsymbol{h}_{\mu\psi,i}^{(j)\intercal} \\ \boldsymbol{h}_{\mu\psi,i}^{(j)} & \boldsymbol{H}_{\psi,i}^{(j)} \end{bmatrix}, \quad \text{for } i = 1,\ldots,n \text{ and } j = 1,\ldots,p, \tag{C18}$$

and vectors

$$h_{\mu,i}^{(j)} \equiv \boldsymbol{\nu}_\mu^{(j)\intercal}\boldsymbol{C}_i^{(j)\intercal}\boldsymbol{C}_i^{(j)}\boldsymbol{\nu}_\mu^{(j)}, \quad \text{for } i = 1,\ldots,n \text{ and } j = 1,\ldots,p. \tag{C19}$$

For each $i = 1,\ldots,n$, the mean vector $\mathbb{E}_q(\widetilde{\boldsymbol{\zeta}}_i)$ and $\mathbb{C}\mathrm{ov}_q(\widetilde{\boldsymbol{\zeta}}_i)$ are defined in (C12). However, $\mathbb{E}_q(\boldsymbol{V}^{(j)})$ and $\mathbb{E}_q(\boldsymbol{H}_i^{(j)})$, $i = 1,\ldots,n$ and $j = 1,\ldots,p$, are yet to be determined. We then have,

$$\mathbb{E}_q(\boldsymbol{V}^{(j)}) = \left[\mathbb{E}_q(\boldsymbol{\nu}_\mu^{(j)}) \quad \mathbb{E}_q(\boldsymbol{\nu}_{\psi_1}^{(j)}) \quad \ldots \quad \mathbb{E}_q(\boldsymbol{\nu}_{\psi_L}^{(j)})\right],$$

where the component mean vectors are defined by (C8). For each $i = 1,\ldots,n$ and $j = 1,\ldots,p$, the expectation of $\boldsymbol{H}_i^{(j)}$, defined in (C18), with respect to the optimal posterior distribution is

$$\mathbb{E}_q(\boldsymbol{H}_i^{(j)}) \equiv \begin{bmatrix} \mathbb{E}_q(h_{\mu,i}^{(j)}) & \mathbb{E}_q(\boldsymbol{h}_{\mu\psi,i}^{(j)})^\intercal \\ \mathbb{E}_q(\boldsymbol{h}_{\mu\psi,i}^{(j)}) & \mathbb{E}_q(\boldsymbol{H}_{\psi,i}^{(j)}) \end{bmatrix},$$

where $h_{\mu,i}^{(j)}$ is defined in (C19) with expected value

$$\mathbb{E}_q(h_{\mu,i}^{(j)}) \equiv \mathrm{tr}\{\mathbb{C}\mathrm{ov}_q(\boldsymbol{\nu}_\mu^{(j)})\boldsymbol{C}_i^{(j)\intercal}\boldsymbol{C}_i^{(j)}\} + \mathbb{E}_q(\boldsymbol{\nu}_\mu^{(j)})^\intercal\boldsymbol{C}_i^{(j)\intercal}\boldsymbol{C}_i^{(j)}\,\mathbb{E}_q(\boldsymbol{\nu}_\mu^{(j)}).$$

Furthermore, $\mathbb{E}_q(\boldsymbol{h}_{\mu\psi,i}^{(j)})$ and $\mathbb{E}_q(\boldsymbol{H}_{\psi,i}^{(j)})$ are defined in (C16) and (C17), respectively.
The multivariate FPCA Gaussian likelihood fragment, summarised in Algorithm 1, is a proceduralisation of these results.

D Additional simulation results

This section contains additional results from the simulation studies.

D.1 Comparison to a model specification with inferred score variances

In this section, we compare the performance of the proposed model formulation (model (8) in the main text) with a variant where the variances of the scores are inferred, using the following hierarchical prior on the scores:

$$\begin{aligned} \zeta_{il} &\sim \mathrm{N}(0,\sigma_l^2), & & i = 1,\ldots,n, \\ \sigma_l^2 \mid a_l &\sim \mathrm{Inverse}-\chi^2(1,1/a_l), \quad a_l \sim \mathrm{Inverse}-\chi^2(1,1/A^2), & & l = 1,\ldots,L, \end{aligned} \tag{D20}$$

where $A = 10^5$. Formulation (D20) allows for a direct account of each component's contribution to the data variability, in contrast to model (8), which fixes the score variance to unity and relies on post-processing steps to estimate the proportion of variability explained by each component. The specification for the other parameters is otherwise identical for the two model formulations; in particular, both versions infer the variances of the spline coefficients representing the latent functions using an iterated inverse-χ^2 prior specification.

In the following simulation, we explore the extent to which estimating both sets of spline coefficient variances and score variances using (D20) enables a more nuanced separation of (i) the variability due to each component, and (ii) the smoothness of the corresponding eigenfunction. To this end, we construct a challenging scenario where less smooth eigenfunctions are associated with lower contributions to the overall data variability: we simulate a dataset with 3 variables – observed at $n_i^{(j)} \in \{30, \ldots, 70\}$ time points for each of $i = 1, \ldots, 200$ subjects – using the following mean function and 4 eigenfunctions:

$$\mu^{(j)}(t) = (-1)^j \sin\{(\pi + j)t\}, \quad \psi_l^{(j)}(t) = (-1)^j \sqrt{2/p} \sin\{(2l-1)\pi t\}, \quad j = 1, \ldots, 3, \quad l = 1, \ldots, 4;$$

hence the periodic eigenfunctions have higher frequencies as l increases, i.e., as the proportion of variance explained by the eigenfunction decreases. The scores ζ_{il} are generated from a centered Gaussian distribution with standard deviation $1/l$, $l = 1, \ldots, 4$, to induce a sharp decrease of the variance explained, and the errors are drawn from a standard normal distribution.

We next perform inference using model (8) and its variant (D20). Figure D1 indicates that both model specifications yield good and virtually identical reconstructions, suggesting that, despite the fixed score variance assumption, model (8) can effectively capture the decreasing smoothness of the eigenfunctions as l increases while accounting for their diminishing impact on data variability. The estimates of the variance explained by the four components, as obtained from the two model formulations, are nearly identical and correspond to: $\approx 74\%$, 17.9%, 5.3% and 2.5% for the 1st, 2nd, 3rd and 4th components respectively, together capturing $> 99\%$ of the total variation. The main difference is computational, with specification (D20) requiring more iterations for convergence, likely due to potential difficulties in maintaining identifiability between the scores and the spline coefficients, whose sets of variances are both inferred. This results in important differences in runtime, namely, the proposed model (8) with fixed score variances converges after 1 min 5 sec (69 iterations), while the variant with inferred score variances (D20) converges after 14 min 33 sec (924 iterations). Thus, the comparable statistical performance supports our preference for the simpler and computationally more efficient formulation (8); the inferred score variance implementation can nevertheless be accessed via an optional function argument in our R package: `https://github.com/hruffieux/bayesFPCA`. It is also important to note that some smoothness is enforced by assumption as we employ O'Sullivan penalised splines to represent the eigenfunctions. As such, neither model specification is intended for settings with highly irregular eigenfunctions.

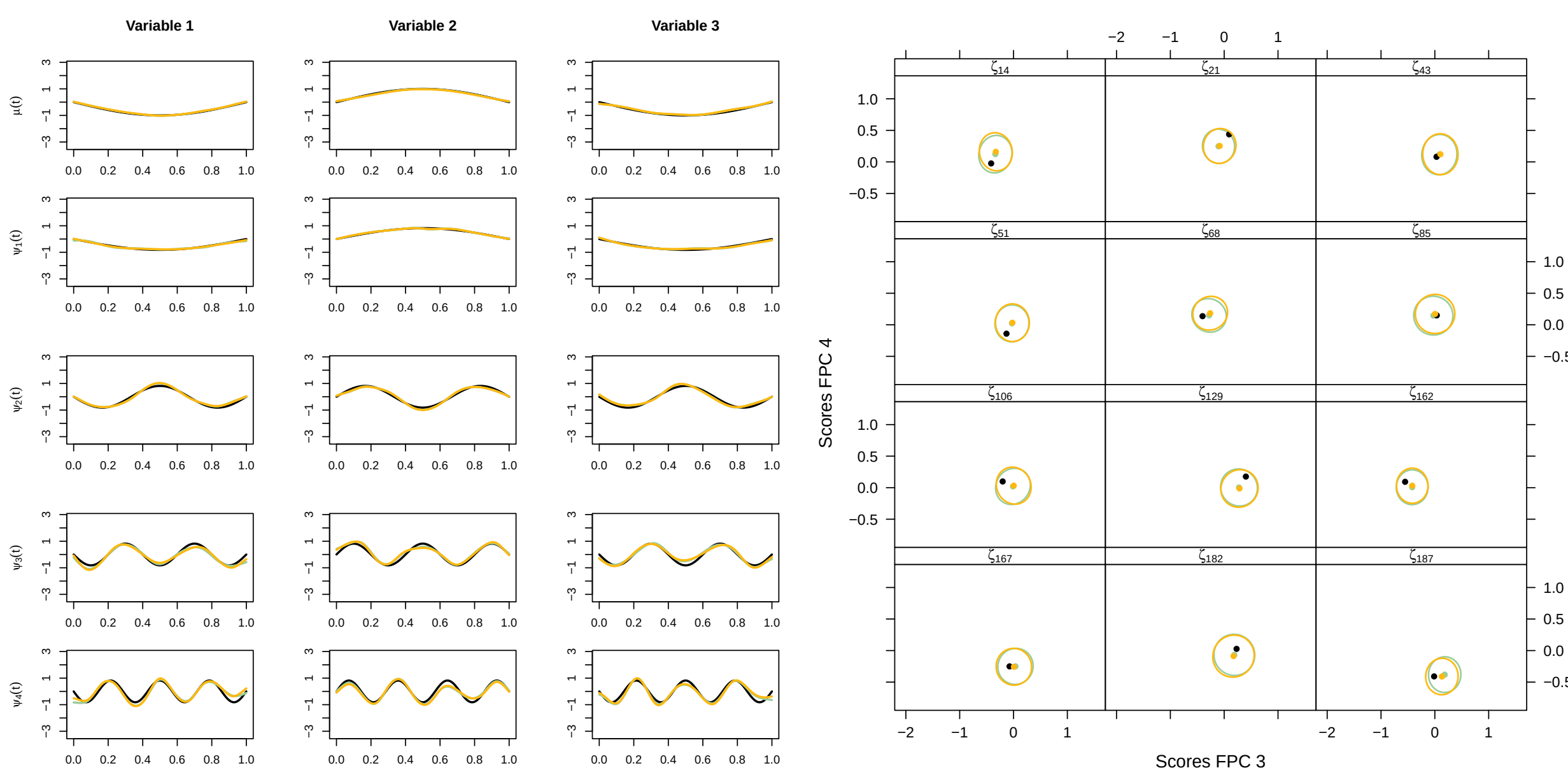

Figure D1: Comparison of the fixed and inferred score variance specifications ($L_{\max} = 10$ and K set with Ruppert (2002)'s rule of thumb), on a problem with $p = 3$ variables observed for $n = 200$ subjects. Left: mean and eigenfunctions simulated (black), estimated with the proposed model (8) (fixed score variances, green) and with modification (D20) (inferred score variances, orange). The green and orange curves overlap. Right: scores for the third and fourth principal components – corresponding to the eigenfunctions with the highest frequencies – simulated (black dots) and estimated with the proposed model (8) (posterior mean, green dots) and with modification (D20) (posterior mean, orange dots) with 95% credible contours, for a random subset of 12 subjects.

D.2 Addendum on the accuracy of the variational algorithm

Figure D2 shows a comparison between the MCMC and MFVB subject trajectories, scores and latent functions, for the problem described in Section 5.2. It indicates strong agreement, similarly as in the comparison between VMP and MCMC estimates.

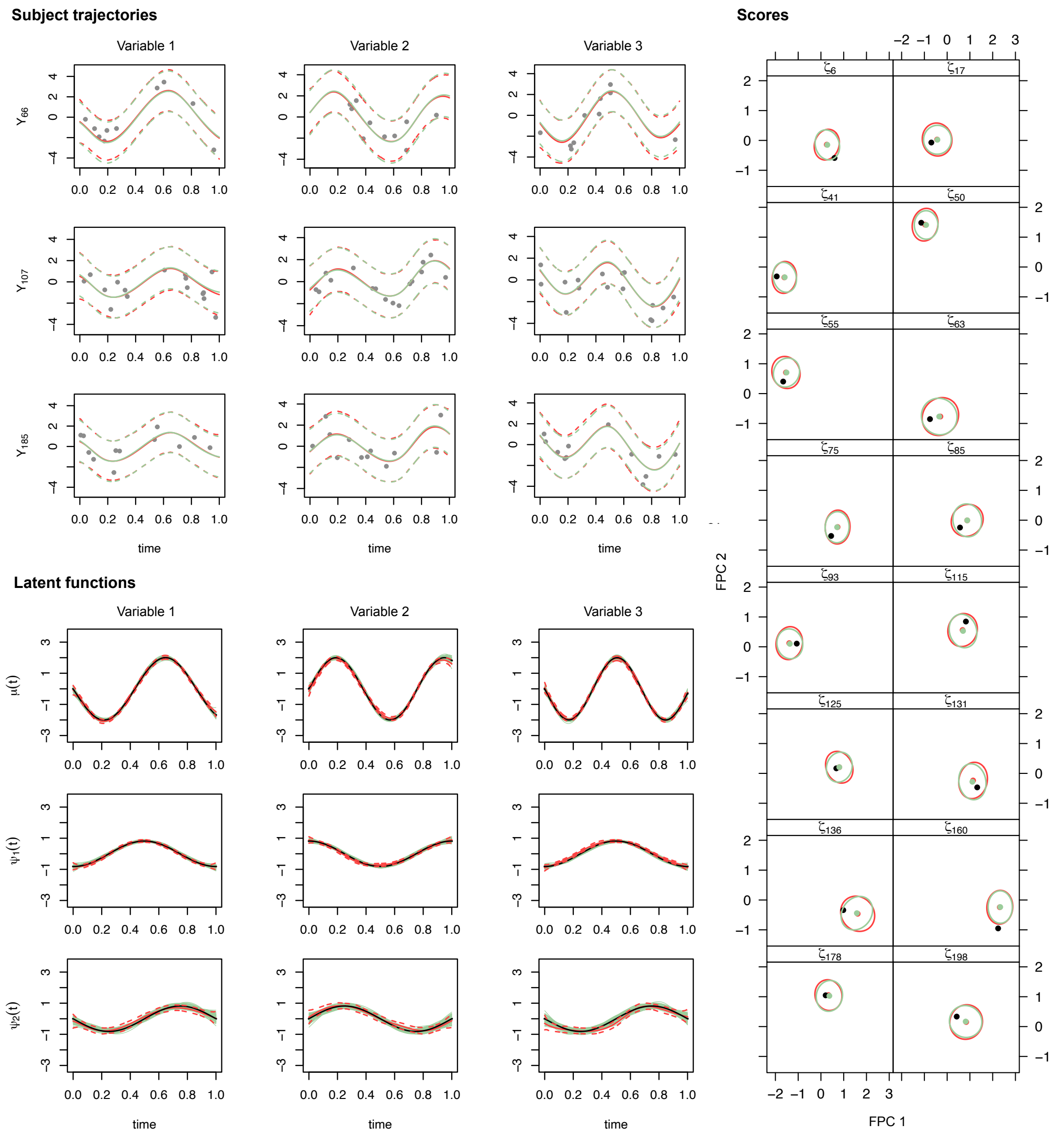

Figure D2: MCMC and MFVB estimates for a problem with $p = 3$ variables observed at an average of 15 time points for $n = 200$ subjects; L is learnt by estimating the PVE with $L_{\text{max}} = 10$, and K is set using the rule of thumb described in Section 4.5 of the main text. Top left: estimated trajectories for a random subset of 3 subjects, with the posterior mean (solid lines) and 95% pointwise prediction bands (dashed). The lines corresponding to MCMC (red) and MFVB (green) inference overlap. Bottom left: mean and eigenfunctions simulated (black) and estimated by MCMC inference (red) with 95% credible bands (dashed) and by MFVB inference (green) for which estimates from 100 replicates are overlaid. Right: scores simulated (black dots) and estimated by MCMC (posterior mean, red dots) inference and MFVB (posterior mean, green dots), with 95% credible contours, for a random subset of 16 subjects.

Figures D3 and D4 show a comparison of the errors on the estimated scores and latent functions for a grid of problems with $n \in \{50, 100, 200, 300, 400, 500\}$, simulating 100 replicates for each. The VMP, MFVB and MCMC errors are very similar, and all decrease as n increases. Additionally, as we expect, when n is small, the variability of the errors

is greater (which is better seen for the scores, whose RMSE is presented on the original scale, rather than for the latent functions whose ISE is shown on the logarithmic scale).

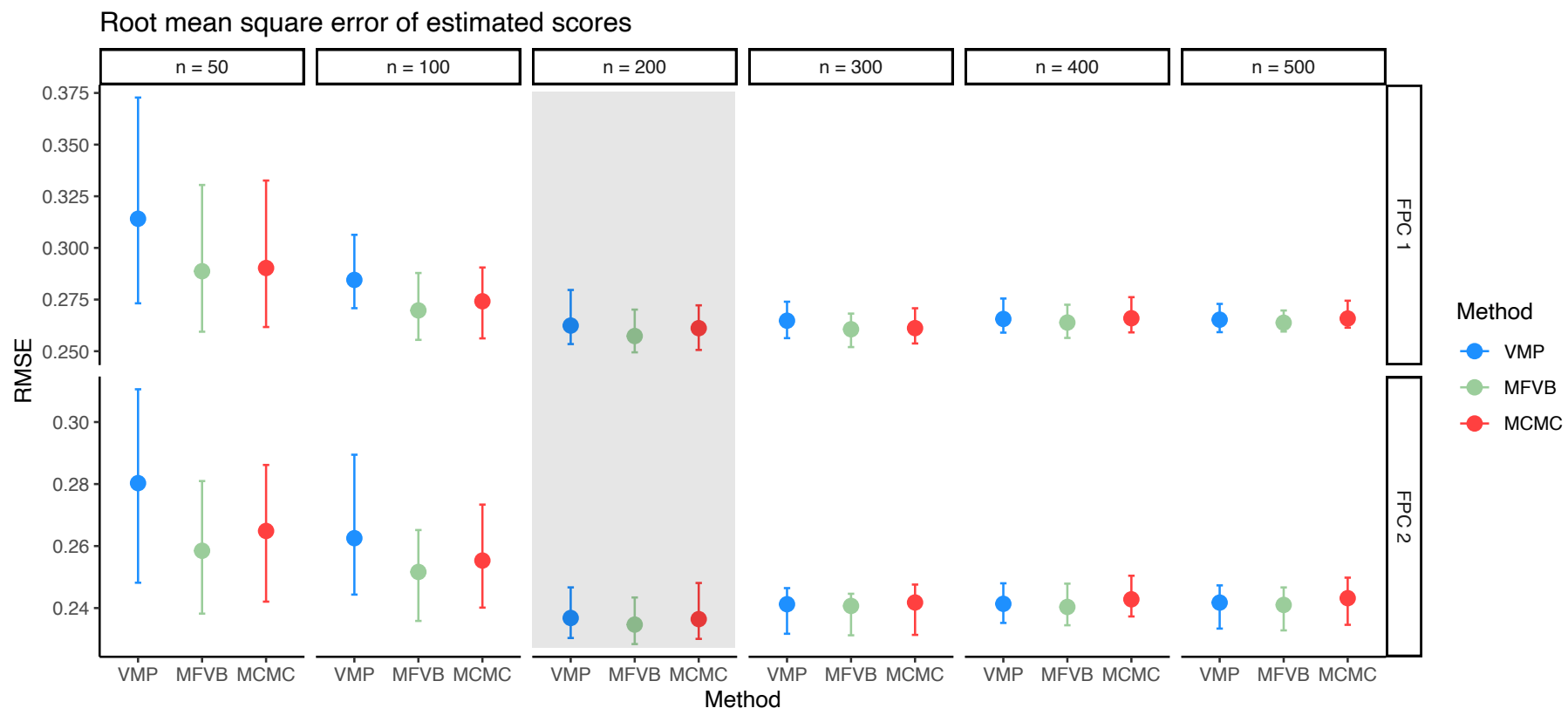

Figure D3: Root mean square error (RMSE) of scores (FPC 1 first row, FPC 2 second row) for a problem with $p = 3$ variables observed at an average of 15 time points for a grid of $n \in \{50, 100, 200, 300, 400, 500\}$ subjects, using VMP (blue), MFVB (green) and MCMC (red) inference. Each scenario is based on 100 data replicates and the scenario shown on Figure 2 is shaded in grey.

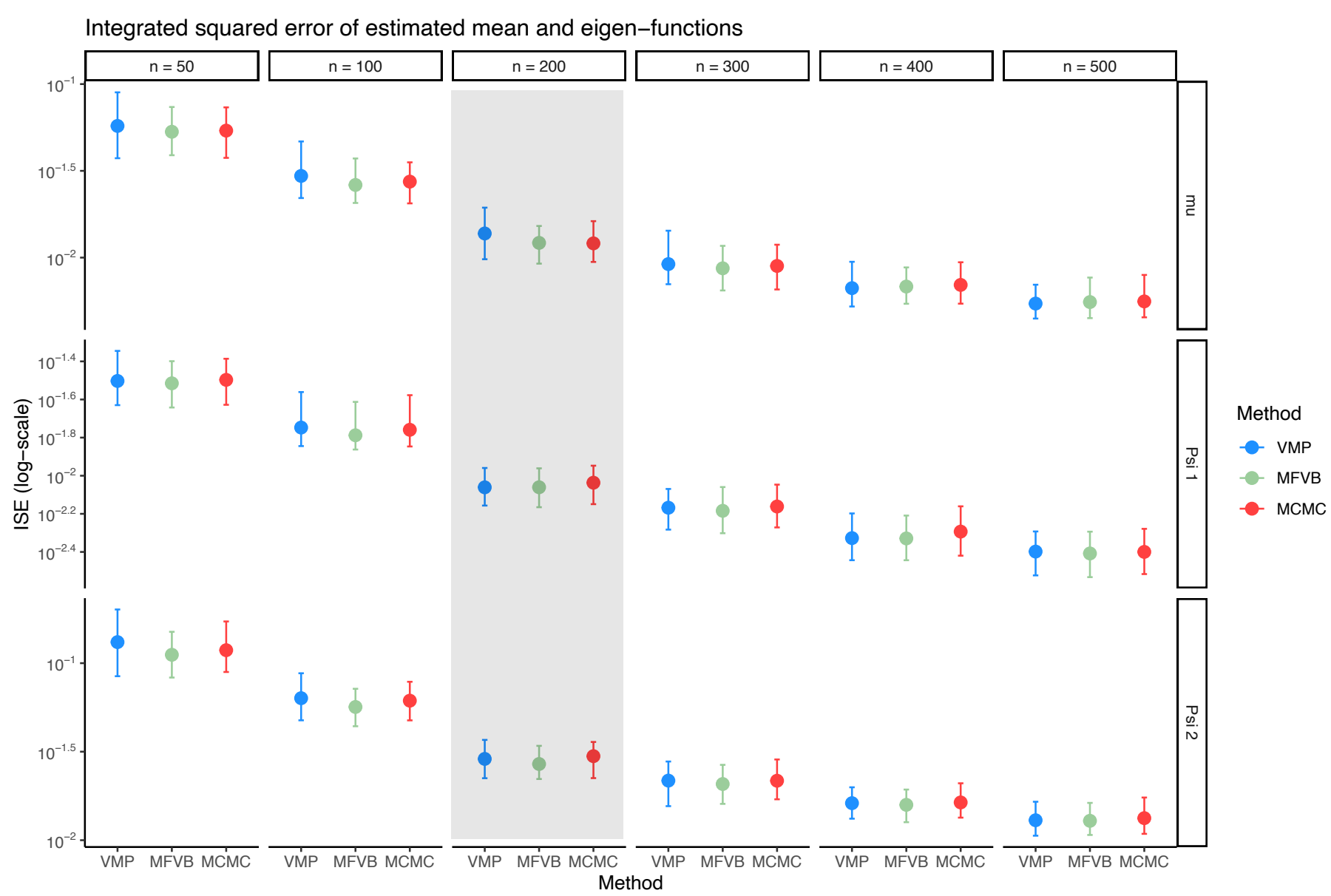

Figure D4: Integrated squared error (ISE) of the mean function (first row) and two eigenfunctions (second and third rows) for a problem with $p = 3$ variables observed at an average of 15 time points for a grid of $n \in \{50, 100, 200, 300, 400, 500\}$ subjects, using VMP (blue), MFVB (green) and MCMC (red) inference. Each scenario is based on 100 data replicates and the scenario shown on Figure 2 is shaded in grey. For each replicate, per-variable ISE are computed and averaged across all p variables.

D.3 Addendum on the selection of K and L

Figure D5 shows the errors on the scores and latent functions achieved by the method when the model is estimated with different choices of numbers of latent functions L and spline basis functions for representing these latent functions K. It suggests that the different approaches to learning K and L, described in Section 4.5 of the main text, result in similar errors, with no sign of overfitting, even compared with random choices $K \in \{5, \ldots, 20\}$ and $L_{\max} \in \{L, \ldots, 10\}$. The data correspond to 100 replicates based on the simulation setup used in Section 5.3.

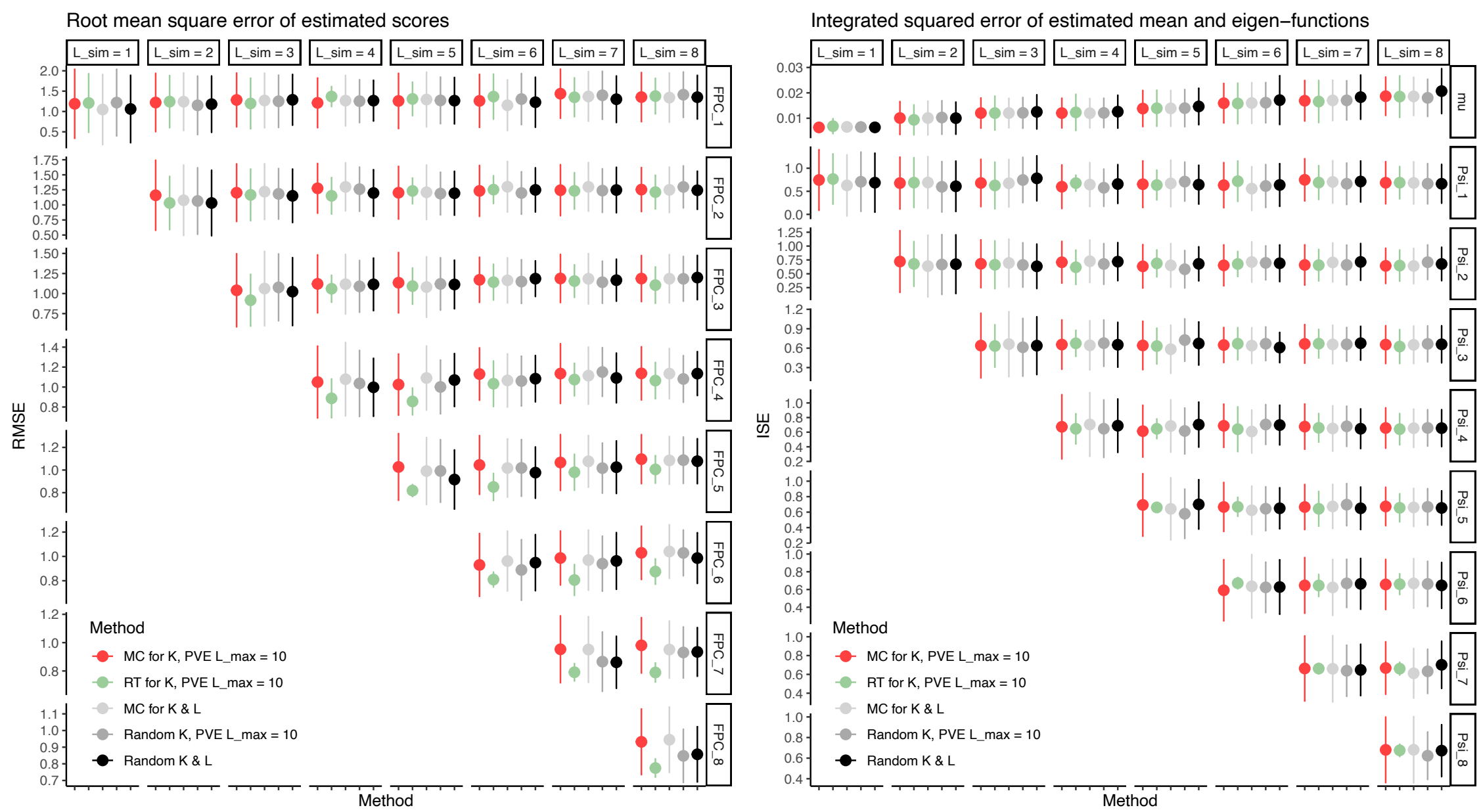

Figure D5: Estimation errors for a problem with $p = 3$ variables, $n = 100$ subjects, 20 observations per subject on average, and with numbers of simulated components ranging from $L = 1$ to $L = 8$ (columns). The procedures for settings K and L are: model choice for K and PVE-based selection for L (red); rule of thumb adapted from Ruppert (2002) for K and PVE-based selection for L (green); model choice for both K and L (light grey); random choice for $K \in \{5, \dots, 20\}$ and PVE-based selection for L (dark grey); random choice for $K \in \{5, \dots, 20\}$ and $L_{\max} \in \{L, \dots, 10\}$. Left: RMSE on the scores. Right: ISE on the mean function (first row) and eigenfunctions (ISE).

D.4 Addendum on the comparison with frequentist multivariate FPCA

Figure D6 compares the estimated scores with the true scores for one replicate of the setting where Happ and Greven (2018)'s method has the highest accuracy according to the simulation results of Section 5.4, namely, with 80 observations per subject and variable, on average. The two methods perform similarly well; note that our approach permits quantifying the uncertainty around the scores thanks to posterior credible ellipses readily available from the estimated VMP posterior distributions.

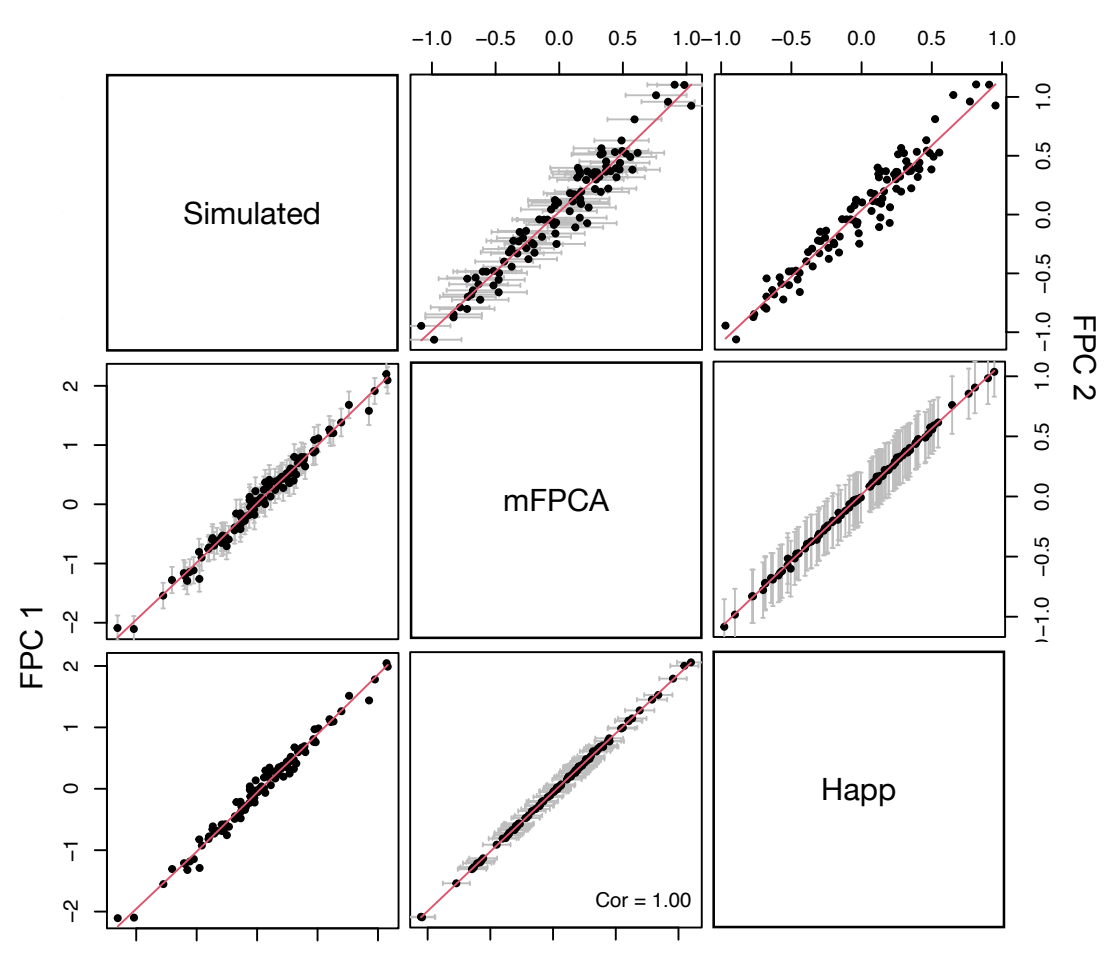

Figure D6: Comparison of scores simulated and estimated with Happ's and our approach for one data replicate of a problem with 80 observations per subject and variable on average. The scores corresponding to the first (resp. second) eigenfunction are shown on the lower (resp. upper) diagonal part. 95% credible intervals are shown for estimates obtained with our approach (grey); such uncertainty quantification is unavailable for Happ's estimates.

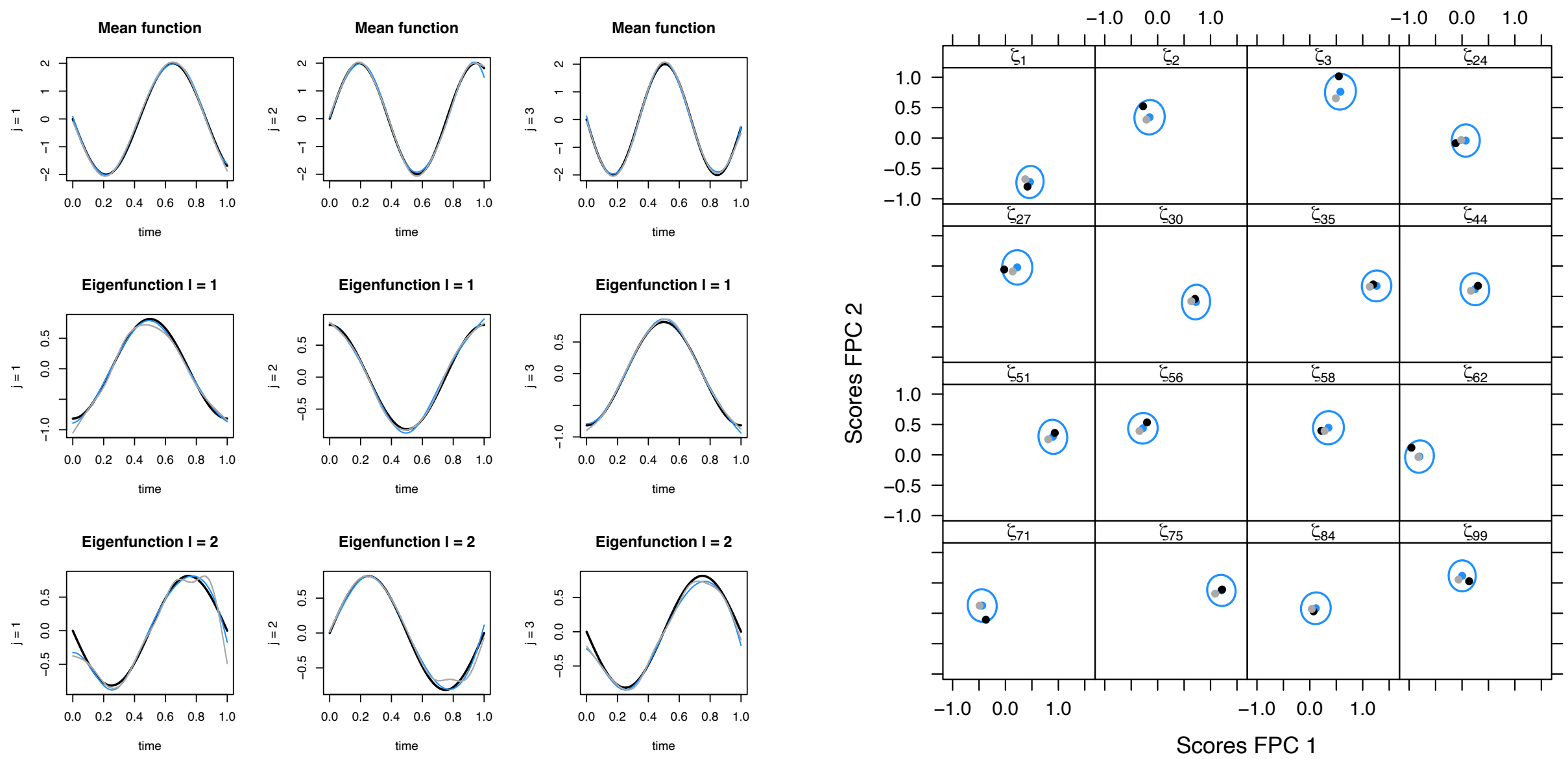

Figure D7: Comparison of FPCA estimates obtained with Happ's and our approaches, for a problem with $p = 3$ variables, $n = 100$ subjects, and an average of 80 observations per variable and subject. Left: mean and eigen- functions simulated (black) and estimated by Happ's (grey) and our (blue) approach. Right: scores simulated (black dots) and estimated by Happ's approach (grey dots) and our approach (posterior mean, blue dots, and 95% credible contours), for a random subset of 16 subjects.

D.5 Addendum on the runtime profiling

Figure D8 shows the runtime of the MFVB and VMP algorithms as a function of the upper bound on the number of components, $L_{\max}$ (see Section 5.7 of the main text for details on the runtime profiling setting).

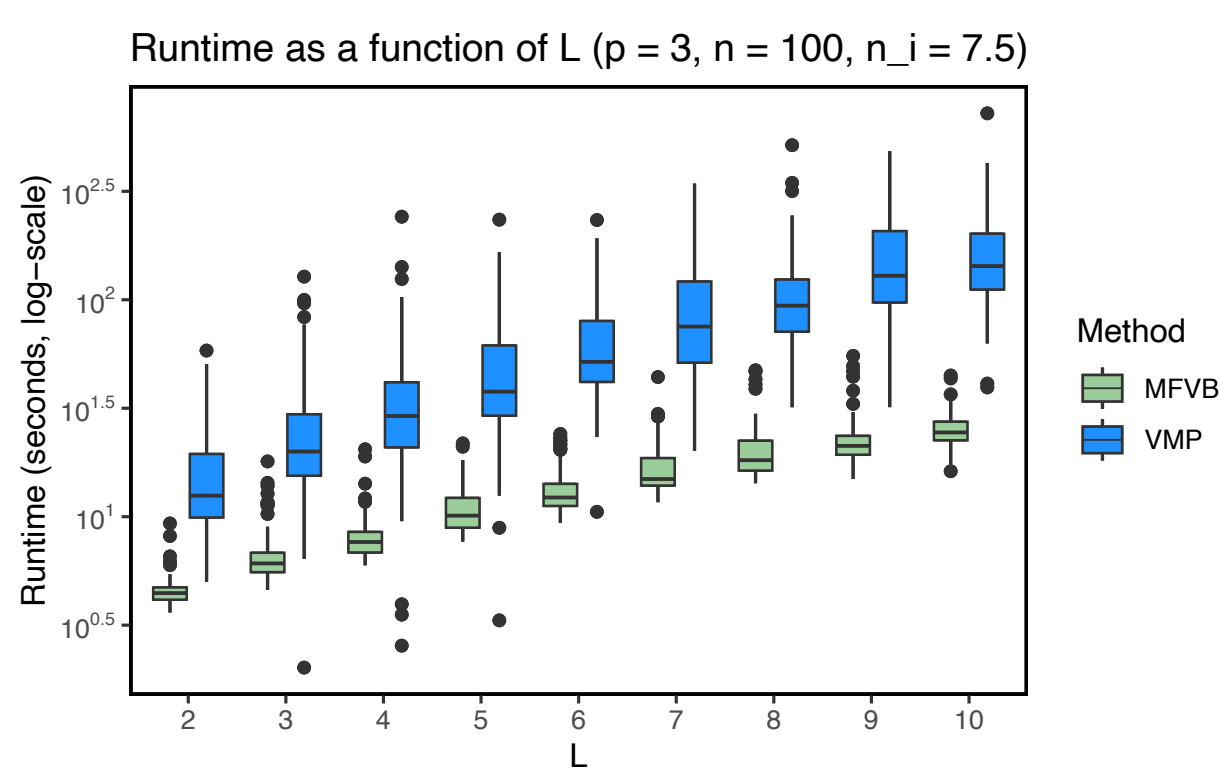

Figure D8: Runtime profiling (on the logarithmic scale) as a function of the number of estimated components, $L_{\max}$, obtained on an Intel Xeon CPU, 2.60 GHz machine. The total time needed for both the inference procedure and subsequent orthogonalisation is shown. Problem with $p = 3$ variables, $n = 100$ subjects, and $n_i = 7.5$ observations per subject on average (100 replicates).

References

J. E. Gentle. *Matrix Algebra*. Springer, New York, 2007.

C. Happ and S. Greven. Multivariate functional principal component analysis for data observed on different (dimensional) domains. *Journal of the American Statistical Association*, 113:649–659, 2018.

L. Maestrini and M. P. Wand. The Inverse G-Wishart distribution and variational message passing. *Australian & New Zealand Journal of Statistics*, 63(3):517–541, 2021.

D. Ruppert. Selecting the number of knots for penalized splines. *Journal of Computational and Graphical Statistics*, 11:735–757, 2002.

M. P. Wand. Fast approximate inference for arbitrarily large semiparametric regression models via message passing (with discussion). *Journal of the American Statistical Association*, 112:137–168, 2017.
